# EEG-based grading of immune effector cell-associated neurotoxicity syndrome

**DOI:** 10.1038/s41598-022-24010-1

**Published:** 2022-11-21

**Authors:** Daniel K. Jones, Christine A. Eckhardt, Haoqi Sun, Ryan A. Tesh, Preeti Malik, Syed Quadri, Marcos Santana Firme, Meike van Sleuwen, Aayushee Jain, Ziwei Fan, Jin Jing, Wendong Ge, Fábio A. Nascimento, Irfan S. Sheikh, Caron Jacobson, Matthew Frigault, Eyal Y. Kimchi, Sydney S. Cash, Jong Woo Lee, Jorg Dietrich, M. Brandon Westover

**Affiliations:** 1 Department of Neurology, Massachusetts General Hospital (MGH), 50 Staniford St. Suite 401, Boston, MA USA; 2 Harvard Medical School, Boston, MA USA; 3 Clinical Data Animation Center (CDAC), MGH, Boston, MA USA; 4 Brigham Young University, Provo, UT USA; 5 Department of Neurology, Brigham and Women’s Hospital (MGH), Boston, MA USA; 6 Dana Farber Cancer Institute (DFCI), Boston, MA USA; 7 MGH Cancer Center for Brain Health, Boston, MA USA; 8 Department of Neurology, Washington University School of Medicine, St. Louis, MO USA

**Keywords:** Cancer immunotherapy, Cancer therapy, Cancer, Haematological cancer, Leukaemia, Lymphoma, Encephalopathy, Neurotoxicity syndromes, Computational neuroscience, Machine learning, Computational biology and bioinformatics, Neuroscience, Biomarkers, Neurology, Oncology

## Abstract

CAR-T cell therapy is an effective cancer therapy for multiple refractory/relapsed hematologic malignancies but is associated with substantial toxicity, including Immune Effector Cell Associated Neurotoxicity Syndrome (ICANS). Improved detection and assessment of ICANS could improve management and allow greater utilization of CAR-T cell therapy, however, an objective, specific biomarker has not been identified. We hypothesized that the severity of ICANS can be quantified based on patterns of abnormal brain activity seen in electroencephalography (EEG) signals. We conducted a retrospective observational study of 120 CAR-T cell therapy patients who had received EEG monitoring. We determined a daily ICANS grade for each patient through chart review. We used visually assessed EEG features and machine learning techniques to develop the Visual EEG-Immune Effector Cell Associated Neurotoxicity Syndrome (VE-ICANS) score and assessed the association between VE-ICANS and ICANS. We also used it to determine the significance and relative importance of the EEG features. We developed the Visual EEG-ICANS (VE-ICANS) grading scale, a grading scale with a physiological basis that has a strong correlation to ICANS severity (R = 0.58 [0.47–0.66]) and excellent discrimination measured via area under the receiver operator curve (AUC = 0.91 for ICANS ≥ 2). This scale shows promise as a biomarker for ICANS which could help to improve clinical care through greater accuracy in assessing ICANS severity.

## Introduction

Chimeric antigen receptor (CAR)-T cell therapy is a breakthrough therapy for relapsed and refractory malignancies with remission rates of up to 93% in patients with relapsed and refractory acute lymphoblastic leukemia (ALL)^1–4^. CAR-T cell therapy is also an effective treatment for chronic lymphocytic leukemia (CLL)^5,6^ and non-Hodgkin lymphoma (NHL)^7^ with the potential to treat an even wider array of cancers and autoimmune conditions^8^. However, CD19 and B-cell maturation antigen (BCMA) directed CAR-T cell therapy can have dangerous side effects, including cytokine release syndrome (CRS) and immune effector cell associated neurotoxicity syndrome (ICANS). ICANS is an acute side effect of CAR-T cell therapy encountered in 20–70% of CAR-T patients^9–13^, that can manifest with altered mental status, headaches, inattention, aphasia, and occasionally with seizures, status epilepticus, and somnolence requiring intubation^14^, and rarely, fulminant cerebral edema and death^14–17^.

Treatment of ICANS is highly dependent on clinicians’ ability to accurately and timely diagnose ICANS and assess its severity. Steroids such as dexamethasone may be beneficial in treating ICANS^18,19^, but prolonged exposure to steroids may diminish the desired effect of CAR-T cell therapy on the underlying cancer^20^. Tocilizumab, which is commonly used to treat cytokine release syndrome (CRS), may also worsen ICANS^21^. A reliable and readily available ICANS biomarker of severity could improve our ability to manage these side effects, provide a cost-saving tool, and improve patient outcomes.

Currently, an objective, accessible, and reliable biomarker of ICANS has not been identified. MRI, cerebrospinal fluid (CSF) profiles, and serum cytokine concentrations have been explored with some success^9^, but the expense and difficulty in obtaining these tests limit their use. Other inflammatory markers, such as C-reactive protein (CRP), lactate dehydrogenase (LDH), and ferritin, have been associated with increased ICANS severity, but lack specificity for ICANS because they are also elevated in CRS^9,14^. Neuroimaging, such as CT and MRI scans, do not typically reveal abnormalities with the exception of severe ICANS resulting in cerebral edema^22,23^. Multivariable models have been effective in assessing the overall risk of ICANS, but do not account for continuous changes in the grade of ICANS on the order of hours^24,25^. Because of these limitations, current procedures for ICANS assessment rely on standardized, frequent bedside examination^17^, which is both subjective and resource intensive.

Prior work suggests that electroencephalography (EEG) has the potential to act as a biomarker for ICANS due to changes in EEG patterns^26^. Patients experiencing ICANS show significant EEG changes including delta and theta slowing and generalized periodic discharges (GPDs), with the severity of these signs correlating to ICANS symptom severity^26,27^. However, findings to date are limited by small sample sizes and are not widely applied in clinical practice. EEG has shown promise as a biomarker in other disease states with similar presentation to ICANS, such as delirium^28–31^. A severity scale for delirium using qualitative EEG features has allowed successful quantification of delirium symptom severity and risk for negative outcomes^31^.

In this study, we examine the significance of a wide range of qualitative (visually assessed) EEG features in ICANS and their correlations with ICANS severity. EEG reports as conventionally written can be difficult for non-neurologists to interpret and often do not influence clinical care outside of excluding seizures, therefore we aimed to develop a data-driven approach to grading EEG findings. We hypothesized that a grading system based on visually apparent features in a patient’s EEG can provide an accurate and objective physiologic measure of the severity of brain dysfunction in ICANS.

## Methods

### Patient cohort

We conducted a dual-center, retrospective, observational cohort study of patients who underwent CAR-T cell therapy at Massachusetts General Hospital (MGH) and Brigham Women’s Hospital (BWH) from May 2016 to November 2020. The study was performed using a waiver of written informed consent under a protocol approved by the Mass General Brigham Institutional Review Board. Use of patient data for this retrospective data analysis was also approved by the Mass General Brigham Institutional Review Board. All the procedures in this study were conducted in accordance with the declaration of Helsinki.

### Clinical data

#### Daily ICANS scores

Daily ICANS scores for each patient were essential to this study. ICANS scores were generated retrospectively via independent chart review by three physicians, PM, SQ and MSF, after being trained by the co-first author CAE, who is a neurologist with neurocritical care training and clinical and research expertise in caring for patients undergoing CAR-T treatment; CAE was also available throughout the chart review process to answer questions. Each reviewer used oncology, neurology, and nursing notes to calculate the Immune Effector Cell-Associated Encephalopathy (ICE) score (0–10) with 10 reflecting a normal exam without deficits. ICE scores capture components of the neurological exam including naming (3 objects; 3 points maximum), orientation (year, month, city, and hospital; 4 points maximum), command following (1 point maximum), attention (subtract from 100 by 10; 1 point maximum), and writing (1 point).

Various substitutions were accepted if a component was not present. For orientation, a patient received a full four points if the chart included “fully alert and oriented” or “oriented × 3” and no other neurological deficits were noted. ICE orientation components could be substituted with date of month, state, current president, or current senator. The attention component of ICE (“subtract from 100 by 10”) was substituted with additional measures of attention including months of the year backward (MOYB), days of the week backward (DOWB), spelling WORLD backward, and subtracting by 7 from 100. A patient received full points for the writing component of ICE if there was no evidence of aphasia or tremor on exam. This was necessary as requesting writing samples did not become routine until the institution of ICE in 2019. For assessment of naming, the full three points were given for, “speech fluent without paraphasic errors” even if three objects were not explicitly recorded, unless the clinician elsewhere noted language deficits.

The ICE score was combined with information from 4 other neurological domains (level of consciousness, motor symptoms, seizures, and evidence of cerebral edema) which together constituted the final ICANS grade (0–4, with 4 being most severe) according to ASTCT guidelines^17^. In cases of discrepancy between reviewers, the average of the two scores was used. Reviewers agreed exactly on 68.85% of cases, disagreed by ± 1 on 25.11% and disagreed by more than 1 on 6.10%.

#### Statistical analysis of cohort characteristics

Data regarding patient characteristics are reported as means (with standard deviation) or medians (with interquartile ranges) and compared using Mann–Whitney U tests. Categorical data are reported as n = count (percentage of total) and compared with chi-square analysis. Significance was defined as *p* < 0.05.

### EEG data

Patients with EEG data on record were identified via chart review of all patients who had received CAR-T cell therapy from 2016 to 2020. Patients were excluded if (1) EEG files could not be located, (2) EEG contained excessive artifact, (3) clinical exam could not be collected and so ICANS score was not available. All EEGs were performed because of concern for ICANS and generally triggered by an alteration in mental status; therefore, most patients in the dataset exhibited neurological symptoms and ICANS at some point during hospitalization. Two patients (1.5%) experienced seizures during their EEG monitoring (Table 1).

**Table 1:** Dataset characteristics.

**Quantitative Data**^**a,b**^**: unique subjects**	Total (n = 120)	ICANS ≤ 2 (n = 42)	ICANS > 2 (n = 78)	*p*-value
Age, years: Mean (SD)	61.1 (11.9)	63.1 (7.3)	60.0 (13.7)	0.3469
Length of stay (days): Median (IQR)	21 (16–29)	18 (15–21)	24 (17–34)	0.0002*
Duration of ICANS > 0 (days): Median (IQR)	10 (6–17)	6 (3–8)	13 (9–20)	0.00001*
Maximum ICANS: Median (IQR)	3 (2–3)	2 (1–2)	3 (3–4)	–
**Categorical data**^**a,c**^**: unique subjects**				
Sex^d^				0.8391
Female: n (%)	40 (33.3%)	13 (31.0%)	27 (34.6%)	
Male: n (%)	80 (66.7%)	29 (69.0%)	51 (65.4%)	
Race^d^				0.5181
Asian: n (%)	4 (3.3%)	0 (0.0%)	4 (5.1%)	
Black: n (%)	3 (2.5%)	1 (2.4%)	2 (2.6%)	
White: n (%)	108 (90.0%)	39 (92.9%)	69 (88.5%)	
Other or Unknown: n (%)	5 (4.2%)	2 (4.8%)	3 (3.8%)	
Ethnicity^d^				0.8294
Hispanic: n (%)	2 (1.7%)	1 (2.4%)	1 (1.3%)	
Non-hispanic: n (%)	114 (95.0%)	40 (95.2%)	74 (94.9%)	
Unavailable: n (%)	4 (3.3%)	1 (2.4%)	3 (3.8%)	
Malignancy^d^				0.1293
DLBCL: n (%)	100 (83.3%)	36 (85.7%)	64 (82.1%)	
PMBCL: n (%)	5 (4.2%)	0 (0%)	5 (6.4%)	
FL: n (%)	5 (4.2%)	4 (9.5%)	1 (1.3%)	
CNS lymphoma	1 (0.8%)	0 (0.0%)	1 (1.3%)	
Other lymphoma	9 (7.5%)	2 (4.8%)	7 (9.0%)	
Secondary CNS involvement	7 (5.8%)	2 (4.8%)	5 (6.4%)	
Aggressive: n (%)	114 (95.0%)	38 (90.5%)	76 (97.4%)	0.2189
Indolent: n (%)	6 (5.0%)	4 (9.5%)	2 (2.6%)	
Medications^d^				
On any AED	115 (95.8%)	40 (95.2%)	75 (96.2%)	0.8108
Levetiracetam	114 (95.0%)	39 (92.9%)	75 (97.4%)	
Lacosamide	3 (2.5%)	0 (0.0%)	3 (3.9%)	
Lorazepam	7 (5.8%)	2 (4.8%)	5 (6.4%)	
Propofol	6 (5.0%)	0 (0.0%)	6 (7.7%)	
One-year post discharge^d^				0.1091
Alive: n (%)	55 (45.8%)	24 (57.1%)	33 (42.3%)	
Deceased: n (%)	43 (35.8%)	10 (23.8%)	31 (39.7%)	
Lost to follow-up: n (%)	22 (18.3%)	8 (19.0%)	14 (17.9%)	

**Quantitative Data**^**a,b**^**: all time points**	Total (n = 315)	ICANS ≤ 2 (n = 156)	ICANS > 2 (n = 159)	*p*-value
EEG feature^e^				
Moderately low voltage	21 (6.7%)	5 (3.2%)	16 (10.1%)	0.0268*
Extremely low voltage	2 (0.6%)	0 (0.0%)	2 (1.3%)	0.4865
Delta slowing < = 1 Hz	35 (11.1%)	3 (1.9%)	32 (20.1%)	0.0000*
Delta slowing 1 < = 2 Hz	115 (36.5%)	53 (34.0%)	62 (39.0%)	0.4190
Delta slowing 2 < = 3 Hz	86 (27.3%)	37 (23.7%)	49 (30.8%)	0.1979
Delta slowing 3 < = 4 Hz	18 (5.7%)	9 (5.8%)	9 (5.7%)	0.8406
Theta slowing 4 < = 5 Hz	99 (31.4%)	36 (23.1%)	63 (39.6%)	0.0024*
Theta slowing 5 < = 6 Hz	96 (30.5%)	56 (35.9%)	40 (25.2%)	0.0514
Theta slowing 6 < = 8 Hz	37 (11.7%)	22 (14.1%)	15 (9.4%)	0.2663
Alpha < = 8 Hz	34 (10.8%)	28 (17.9%)	6 (3.8%)	0.0001*
Alpha 8 < = 9 Hz	22 (7.0%)	19 (12.2%)	3 (1.9%)	0.0008*
Alpha > = 9 Hz	24 (7.6%)	17 (10.9%)	7 (4.4%)	0.0500*
PDR present	42 (13.3%)	42 (26.9%)	0 (0%)	0.0000*
GRDA	8 (2.5%)	3 (1.9%)	5 (3.1%)	0.7408
GPDs	16 (5.1%)	2 (1.3%)	14 (8.8%)	0.0054*
LRDA	3 (1.0%)	2 (1.3%)	1 (0.6%)	0.9868
LPDs	5 (1.6%)	1 (0.6%)	4 (2.5%)	0.3788
Intermittent brief attenuation	4 (1.3%)	0 (0%)	4 (2.5%)	0.1361
Burst suppression	1 (0.3%)	0 (0%)	1 (0.6%)	0.9924
Seizures^f^	10 (3.2%)	0 (0%)	10 (6.3%)	0.0042*

Patient Characteristics according ICANS grade ≤ 2 as compared to > 2 (severe ICANS).

^a^Dataset included 135 individual subjects who received CAR-T cell therapy.

^b^Quantitative data are shown as medians (IQR) or means (SD) and compared using Mann–Whitney U tests.

^c^Categorical data are shown as *n* = counts (percent) and compared using Chi-Squared tests. The significance level was set at *p* < 0.05 and is indicated by *.

^d^Demographic data (age, sex, race, and ethnicity), subtype of malignancy, and death at one-year post discharge are based on information extracted from the electronic health record.

^e^EEG features were extracted from 15 s images manually.

^f^Seizures were not included as an input to the model due to their direct correlation with clinical ICANS scores (if present, ICANS is an automatic 3 or above).

EEGs performed as part of clinical care were recorded with Ag/AgCl scalp electrodes using the standard international 10–20 electrode placement by qualified EEG technicians. Recordings were formatted as a bipolar montage and filtered using normal 60 Hz notch filters and bandpass filters (0.5–40 Hz). EEGs were split into 15 s segments and flagged for the presence of artifact. For each 24-h period of continuous EEG per patient, a single segment was selected by choosing segments free of significant artifact, near in time to morning nursing assessments, where possible with clear signs of wakefulness (e.g. eye blinks) to select EEGs representing patients’ most awake state. When clear signs of wakefulness were not present, we excluded segments with normal sleep microarchitecture (i.e. sleep spindles, K complexes, or vertex waves). This was done because features that are abnormal in a “maximally awake” patient with encephalopathy (e.g. generalized irregular theta or delta slowing) can be normal in sleep. The 15 s length of segments were selected for our study because this is the typical window size for clinical review of EEGs and scoring longer segments would be more labor intensive. We considered this duration sufficient as qualitative changes in EEG are typically stable in our experience unless viewed on a scale of 30 min or more, and ICANS grade tends to evolve slowly in most patients (Table 1).

### Feature selection and labelling

**Figure 1:**
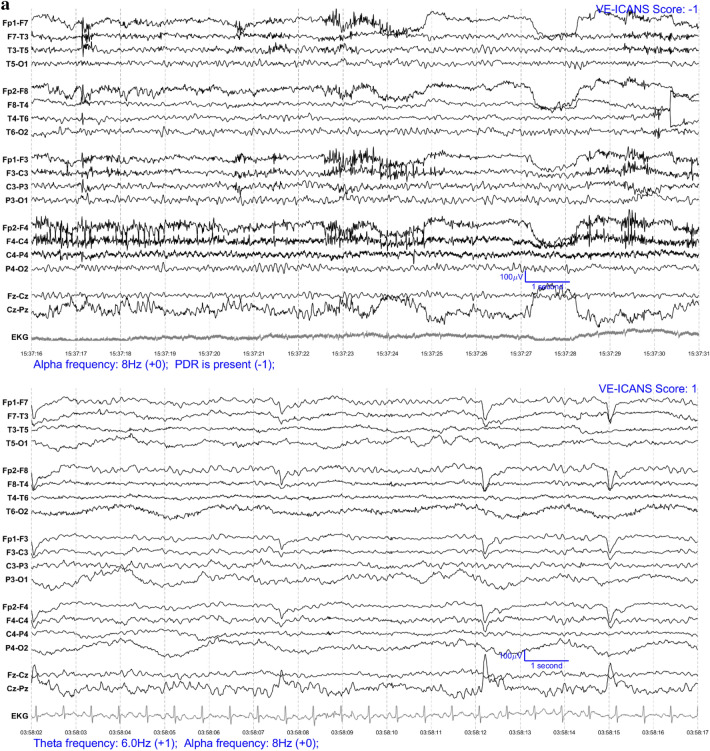

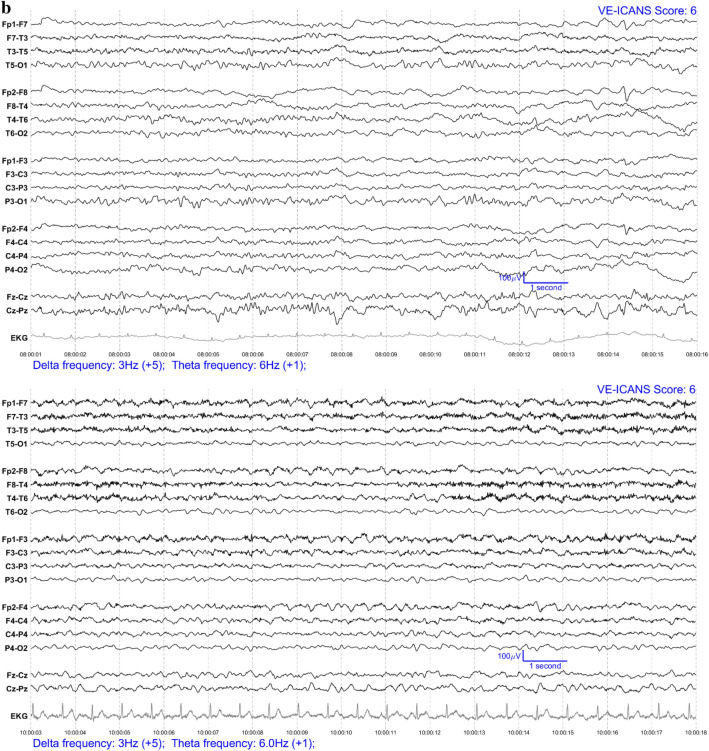

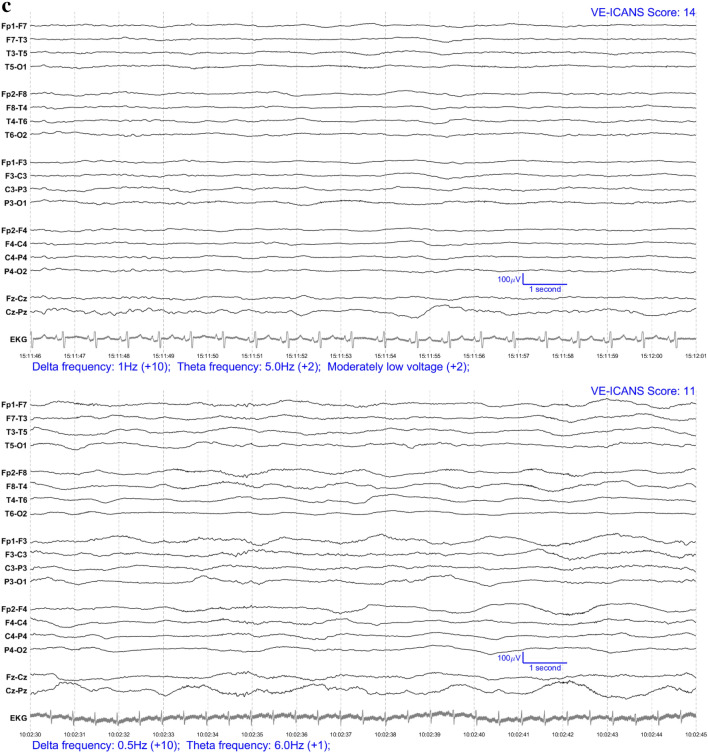
EEG examples from patients who recently received CAR-T therapy (in Bipolar Montage format). The VE-ICANS score given to each image by the VE-ICANS model is shown in the upper right corner. The graded features that contributed to that score are shown in the bottom left along with their point values. (**a**) Example EEGs for mild/no ICANS. (**b**) Example EEG for moderate ICANS severity. (**c**) Example EEG for high ICANS severity.

Each EEG segment was converted into an image with one image per patient per day (see Fig. 1) and were completely anonymized. These images were ranked by three neurologists (two epileptologists (BW, FN) and one neurologist (CE) with research training in EEG analysis) through visual assessment according to severity of encephalopathy to attempt to leverage the implicit knowledge that an expert has in what makes one EEG worse than another. The three scorers discussed these images to develop a set of EEG features (see Table 2) intended to encompass all aspects of the EEG that experts (implicitly) were likely to have relied on when ranking the EEGs.

**Table 2:** Image grading scheme.

1. Clearly discernable delta oscillations present? If so what frequency? (0.5–4.0)
2. Clearly discernable theta oscillations present? If so what frequency? (4.5–7.5)
3. Clearly discernable alpha oscillations present? If so what frequency? (8–12)
4. Beta oscillations present?
5. Posterior Dominant Rhythm (PDR) present?
6. Signs of being awake?
7. Generalized Rhythmic Delta Activity (GRDA) present?
8. Generalized Periodic Discharges (GPDs) present?
9. Lateralized Periodic Discharges (LPDs) present?
10. Moderately Low Voltage (< 20 uV) present?
11. Intermittent Brief Attenuation present?
12. Nonconvulsive Status Epilepticus (NCSE)?
13. Low voltage: Extreme/Electrocerebral Silence (ECS)?
14. Burst suppression present?
15. Unreactive EEG?

The grading scheme used in visually grading the EEG images. Features 12–15 represent severe encephalopathy and were automatically assigned worst ICANS severity. Features were translated to a binary mapping for model training by subdividing slowing frequencies into 1 Hz ranges (e.g. Delta frequency 0–1 Hz). Some of these were recombined after training if coefficients were the same (see Table 3).

**Table 3:** Visual EEG-based ICANS (VE-ICANS) grading system.

EEG Feature	Score
Delta frequency 1 Hz or less	+ 10
Delta frequency 1–2 Hz	+ 6
Delta frequency 2–3 Hz	+ 5
Delta frequency 3–4 Hz	+ 3
Moderately low voltage (< 20 μV)	+ 2
GRDA (Gen. rhythmic delta activity)	+ 2
GPDs (Gen. Period Discharges)	+ 2
Theta frequency 4–5 Hz	+ 2
Theta frequency 5–8 Hz	+ 1
Alpha frequency > 9 Hz	−1
PDR present	−1
NCSE (Nonconvulsive Status Epilepticus) Low voltage: Extreme/ECS (Electrocerebral Silence) Burst suppression@Unreactive EEG	Maximal score

A table showing the coefficients for each feature in the VE-ICANS model. A patient’s VE-ICANS score is the sum of all features present in their EEG. Positive scores indicate a feature with a positive correlation to ICANS severity. Features with the highest values are the most significant features in grading ICANS severity and contribute the most to the VE-ICANS score.

All of the 15 s images were then labelled using the final feature set by a senior epileptologist (MBW) according to the grading scheme in Table 2. In addition, three epileptologists independently graded a small set of images (n = 10). We quantified inter-rater agreement by mean and standard deviation of the resulting VE-ICANS scores, and the percent of scores with differences of 0, 1, or more points.

### Learning-to-rank algorithm

Analysis was performed using a pairwise learning-to-rank (LTR) machine learning algorithm^31^. The LTR model was trained using the visually assessed features present in each image along with the ICANS score generated for that day. The model learns coefficient values for each feature by predicting for each pair of images A and B, whether the features from A represents more severe ICANS than the features from B. By comparing all pairs of images in this way, a relatively small dataset of 315 images becomes a much larger training set of ~ 40 k pairs. Pairs with equal ICANS values were ignored. The model uses logistic regression to find input feature coefficients which maximize the number of pairs ranked correctly according to their ICANS values. The final VE-ICANS score for each image was the sum of the coefficients for the features present in that image.

To avoid overfitting and to reduce the effects of collinearity, we imposed several a priori constraints on the model based on medical domain knowledge. These constraints included (1) ElasticNet penalty: a regression regularization method that reduces overfitting by linearly combining the L_1_ and L_2_ penalties of the LASSO and ridge methods in the penalty function^32^; (2) integer constraint: coefficients were constrained to be integers so that they could more easily be used by practitioners as point values in grading an EEG; (3) sign and severity constraints: medical domain knowledge was applied in constraining abnormal EEG features to have positive coefficients (point values) and normal EEG features to have negative coefficients; certain patterns of severe encephalopathy, which appeared rarely in the dataset, were set a priori to receive the maximal possible score; and (4) ordinal constraints: coefficients for all slowing frequencies were constrained so that lower frequency (more severe) slowing had coefficients larger than or equal to the coefficients for higher frequency (less severe) slowing. A minimum of 5 instances of each feature was required across the dataset to be considered for inclusion in the feature set used to train the model. This meant that rare features (low representation in the dataset) were not used in model development.

### Model training

Model training consisted of five-fold nested cross validation (CV). The outer CV loop split the dataset into five folds in a stratified manner to maintain equal distribution of ICANS values and to keep all rows from a single patient in the same fold to avoid information leakage across folds. Four folds were used to train the model (training set) while one fold was used to estimate out-of-sample performance (testing set). The final out-of-sample performance was estimated using the average of the predictions on the five testing folds. Out of sample performance was measured using the Pearson’s correlation coefficient between predicted scores and ICANS scores. 95% confidence intervals were generated through bootstrapping 1000 times. The area under the receiver operator curve (AUC) was used to assess the ability of the final model to discriminate levels of ICANS severity (i.e. ICANS 0 vs. ≥ X). We also compared our VE-ICANS model with another model (VE-CAM-S) developed to measure the severity of encephalopathy in the general hospitalized patient setting, as opposed to our VE-ICANS grading scale which is designed to specifically assess ICANS in patients who have undergone CAR-T treatment^31^.

### Ethics approval and consent to participate

The study was performed using a waiver of written informed consent under a protocol approved by the Institutional Review Board. Use of patient data for this retrospective data analysis was approved by the Mass General Brigham Institutional Review Board, reference number 2013P001024.

## Results

### Dataset characteristics

In this study, 136 patients were included, representing the full range of ICANS (0–4). The average patient had 3 days of EEG recording with values ranging from 1 to 14 days. From this, 427 images were generated. 112 (26%) of these were excluded based on the criteria outlined in the methods section. Of the remaining 315 images, 159 (50%) were from patient days with severe ICANS (3–4), and 156 (50%) were from days with mild ICANS (1–2). These 315 images represent 120 (88.2%) of the patient cohort. Table 1 provides a full description of patient and EEG characteristics.

### The VE-ICANS model

**Figure 2:**
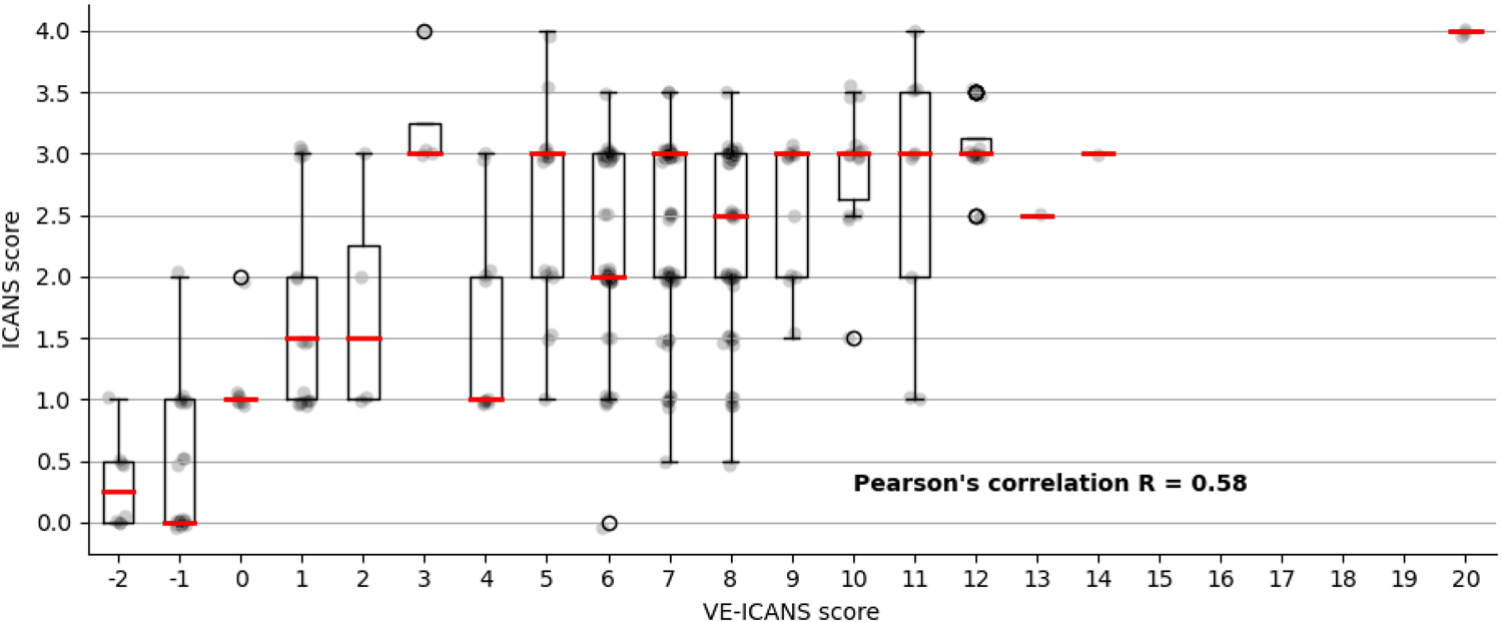
Distribution of predicted scores (VE-ICANS) versus ICANS scores. The VE-ICANS scores are highly correlated with ICANS (avg. value of 0.58 across 1000 bootstraps with 95% CI 0.47–0.66). For each level of VE-ICANS (x-axis), a box plot shows the distribution of ICANS scores (y-axis). The box plots use traditional ranges with 50% inside the box and whiskers extending to points within 1.5 × the inter-quartile range. Means at each level are shown in red. The semitransparent points (grey) show the value of VE-ICANS for each datapoint in the dataset with darker areas indicating a greater concentration of points.

**Figure 3:**
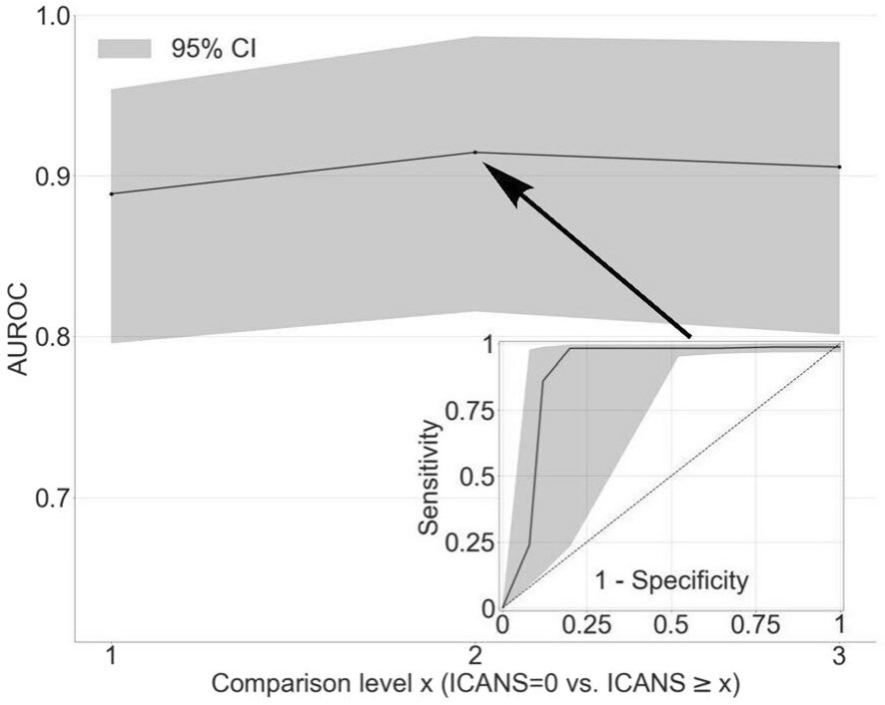
Area under the receiver operating curve for various comparison levels of ICANS. Plot showing how the VE-ICANS grading scale performs in discriminating patients without neurotoxicity (ICANS = 0) from those with neurotoxicity (ICANS ≥ x), for various levels of neurotoxicity, x. Here, discrimination is measured by area under the receiver operating characteristic curve (AUC). The shading indicates the 95% confidence intervals. The inset figure shows the receiver operating curve for discriminating ICANS = 0 compared to ICANS ≥ 2.

For each EEG image, the LTR model produced a physiological assessment of ICANS severity: the Visual EEG based-ICANS score (VE-ICANS). VE-ICANS scores were strongly correlated with clinical ICANS grades with a Pearson’s correlation R = 0.59 (95% CI 0.47–0.66, see Fig. 2). For all levels of comparison ICANS = 0 versus ICANS ≥ X (Fig. 3), the model showed high levels of discrimination with average area under the curve across all levels greater than 0.9. The area under the curve shows excellent discrimination with AUC of 0.91 (95% CI of 0.82–0.99) for discrimination of ICANS ≥ 2 (Fig. 3).

By comparison, correlation of ICANS with scores from a previously established model for delirium severity, VE-CAM S, was moderate (R = 0.42).

### EEG features with high predictive value

**Figure 4:**
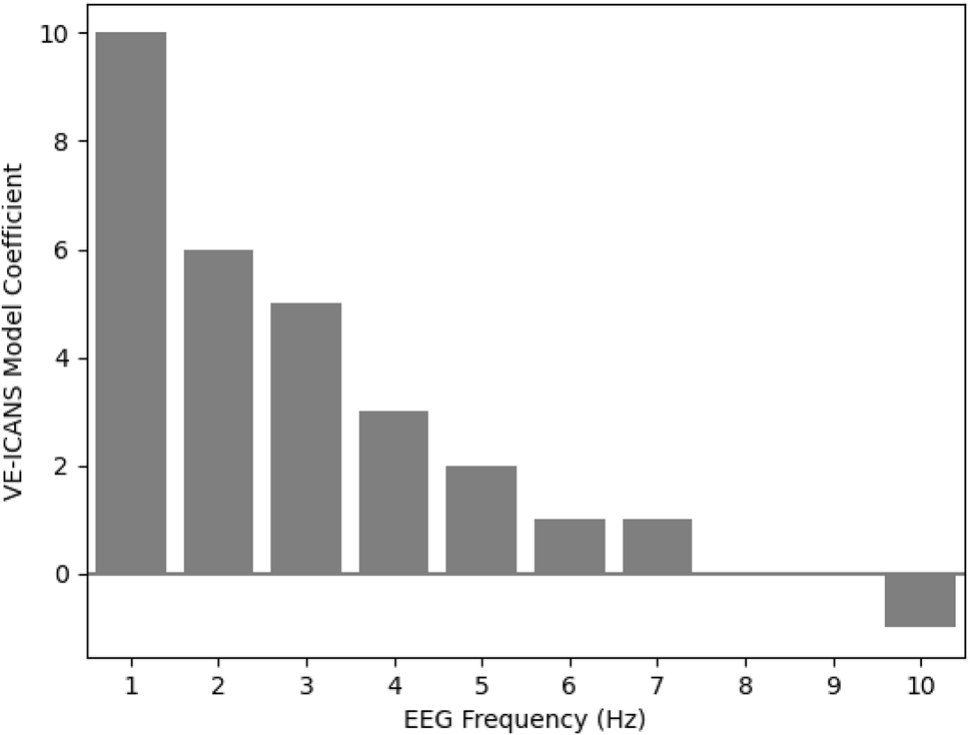
VE ICANS coefficients for each frequency (Hz) of slowing. A bar plot of the assigned coefficient values (y-axis) in the VE-ICANS model of various degrees of slowing (x-axis) showing how incremental increases in slowing are given more weight in prediction of ICANS severity. “Slowing” is defined as an EEG frequency less than 8 Hz.

**Figure 5:**
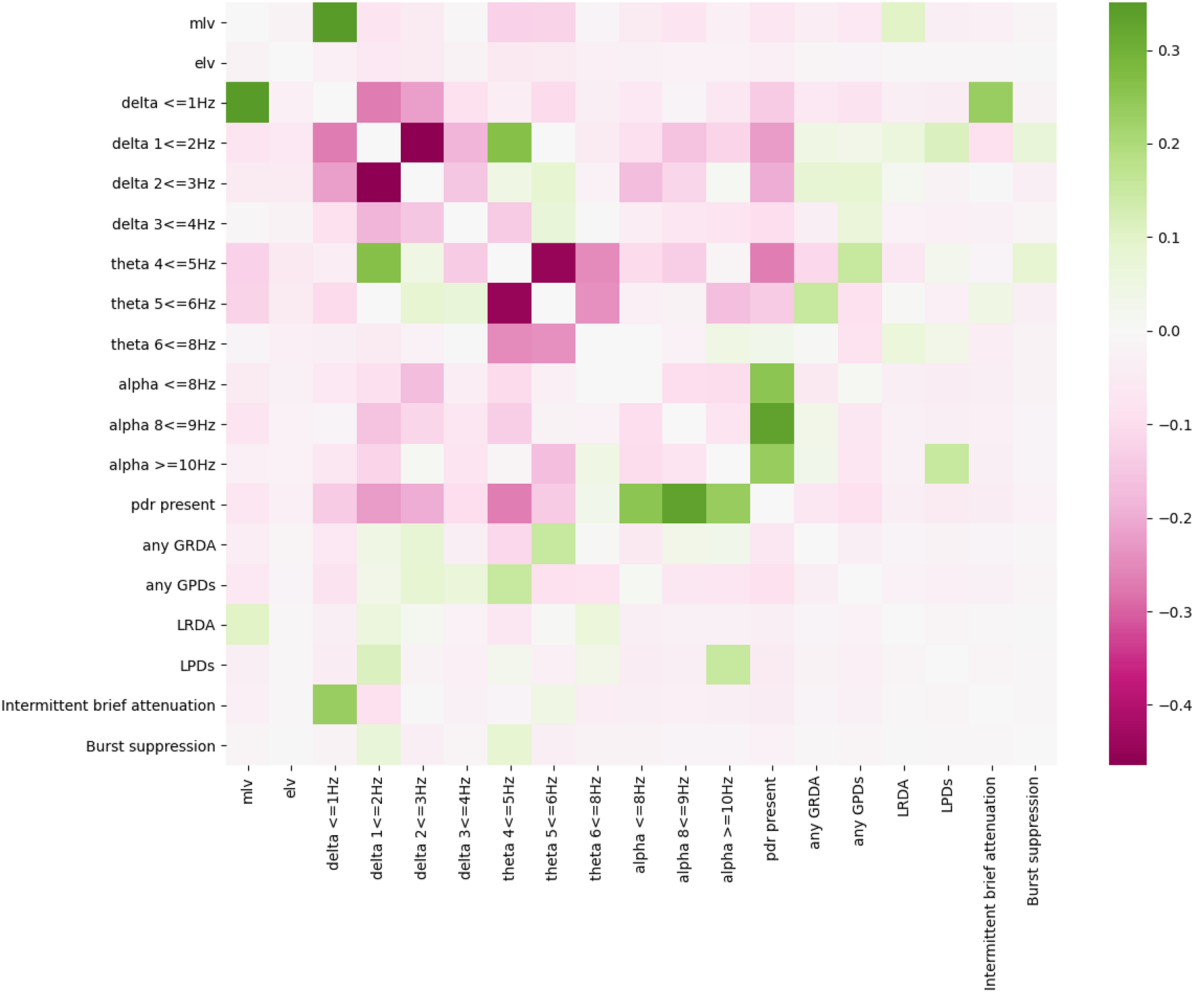
Spearman’s correlations between all EEG features in the model. A heatmap showing Spearman’s correlations between all input features of the VE-ICANS model (*green*, positive correlation vs. *pink*, negative correlation). Features with positive correlation were often present in the same image.

Each feature included in the final model was given an integer value representing its contribution to the overall score. Coefficient values ranged from − 1 (for positive features such as an alpha frequency of > 9 Hz or a discernable PDR) to + 10 (for delta frequencies of 1 Hz or less). Slowing features were split (see Table 2) into individual frequencies and represent a significant portion of the features in the model (Fig. 4). Delta slowing frequencies show the greatest range of values with 3–4 Hz having a score of 3 while delta slowing in the 1–2 Hz range has a score of 6 and less than 1 Hz has a score of 10. Theta slowing frequencies show less variation but are also significant with frequencies between 5 and 8 Hz receiving a score of 1 and 4–5 Hz a score of 2. Theta slowing at 4–5 Hz is equal to other features in the model including GPDs, GRDA and moderately low voltage (< 20 μV) which also have scores of 2. Note that these scores must be interpreted together; the higher score associated with delta slowing compared with GPDs does not mean that GPDs are “less bad”, because these features (and others) frequently co-occur (Fig. 5) and are counted together to produce the total score.

### Inter-rater reliability

A small trial using a random subset of images (n = 10) showed high agreement between VE-ICANS scores based on three epileptologists who graded images independently (mean absolute deviation = 0.75, STD of absolute deviation = 0.72, percent with difference of 0, 1, or ≥ 2: 70%, 23%, 7%).

## Discussion

In this study, we developed an EEG-based grading scale that strongly correlates with ICANS (R = 0.58 [0.47–0.66], see Fig. 2) and discriminates well between levels of ICANS (AUC 0.91 [0.82–0.99], see Fig. 3). This scale shows promise as an objective diagnostic biomarker of ICANS and is readily amenable to implementation, given its qualitative features are easily identified through visual inspection.

Although prior investigations have described qualitative EEG findings in moderate to severe ICANS^33,34^, our study is the first to incorporate these features into a model of ICANS severity, whose coefficients reveal the importance of each EEG finding in predicting ICANS. The lower EEG frequencies (i.e. < 8 Hz), reflecting slowing of the EEG, and, in particular, EEG < 3 Hz were the most predictive features in the model. Furthermore, the granularity of slowing (Fig. 4), on the order of 1 Hz rather than between standardized frequency bands (delta vs. theta), enhanced performance of the model, as compared to application to the same dataset of VE-CAM-S, which is an EEG based model of delirium that does not delineate degree of slowing within frequency bands. This finding indicates greater clinical relevance to the granularity of slowing than previously recognized, even though EEG reports often do not provide such information in their descriptions.

In addition to highlighting important EEG features, VE-ICANS coefficients also indicate that previously identified EEG findings in ICANS may carry less predictive power across all patients with ICANS. GPDs proved less prominent in VE-ICANS than slowing, likely due to their rare occurrence in our dataset, even though GPDs were one of the first EEG findings to be described in ICANS, likely due to their salience in severe ICANS cases^26^. Other features, such as LPDs and LRDA, were not retained in our model due to LASSO regularization rather than collinearity with other features, even though these findings have also been observed in other studies of ICANS. This may reflect underpowering of our dataset for these features, or may indicate that such features are only relevant in certain subtypes of ICANS, particularly since LPDs and LRDA have been associated with focal neurological findings^33^.

Although VE-ICANS outperformed a previously published delirium model, VE-CAM-S, when applied to our dataset, the moderate performance of VE-CAM-S (R = 0.42) supports similarities in neurophysiology between delirium and ICANS. Slowing has been identified as a prominent, highly sensitive feature in delirium^30,31^, similar to our model’s finding in regard to ICANS. Moderately low voltage also has similar importance in both the delirium and ICANS models. However, the most heavily weighted feature in the delirium model, intermittent brief attenuation, was not retained in VE-ICANS, likely due to its high correlation with another prominent, retained feature, delta < 1 Hz (Fig. 5).

There are several limitations to this study. ICANS scores were fundamental to developing our model. However, the retrospective scoring of ICANS likely introduces some noise into this gold standard. Moreover, ICANS is dynamic, sometimes changing on the order of hours, and we did not have timestamps for the exams from which we determined ICANS scores. Future prospective studies could provide more standardized ICANS scores. Grading a larger portion of the EEG, rather than only the 15 s segments used, might reveal longer-term patterns that carry additional information about ICANS (for example, patterns related to sleep or sleep disturbances). Such analyses are more labor intensive but may be facilitated by automated quantitative approaches. EEGs in the current study were graded by a single expert. Future studies will need to investigate inter-rater reliability and, before adoption into clinical use, should develop standardized materials for teaching and assessing accuracy in using VE-ICANS. A larger cohort may also contain more features predictive of ICANS scores which were not prevalent enough in our dataset to be included in the model. Due to EEG only being recorded for patients with altered mental status, not all patients who received CAR-T could be included. Nearly all patients in the study received an anti-epileptic drug, primarily levetiracetam. Levetiracetam is routinely used in the outpatient setting and does not typically produce visually apparent changes in the EEG (except modestly reducing interictal epileptiform discharges^35,36^). A minority of patients (N = 6), all with severe ICANS, received propofol, which strongly influences the EEG, inducing delta and theta slowing and, at high doses, burst suppression. Future analyses could incorporate quantitative EEG features and EEGs from patients who received CAR-T therapy but did not develop ICANS, as well as examine the prognostic ability of the EEG.

## Conclusions

In summary, the VE-ICANS grading scale facilitates detection of ICANS based on a set of readily visualized, qualitative EEG features and supports the potential of EEG as a biomarker for ICANS. Although non-epileptiform EEG findings are often ignored in EEG reports, the VE-ICANS harnesses this previously untapped information, thereby enhancing the utility of EEG beyond simply excluding seizures. Due to the scale’s reliance on qualitative rather than quantitative features, VE-ICANS could be easily incorporated into standard EEG interpretation among this patient population. In turn, VE-ICANS could boost the interpretability and usefulness of EEG for non-neurologists, who are most often caring for these patients. Such a physiologically based biomarker for ICANS severity could improve clinical care by enhancing detection and tracking of ICANS, thereby enabling more effective interventions for ICANS and increasing implementation of CAR-T cell therapy for more indications and in additional care centers.

## Data Availability

The datasets used and/or analyzed during the current study are available from the corresponding author on reasonable request. They are currently being de-identified and in the future will be available here: https://github.com/mghcdac/VE-ICANS.

## Abbreviations

CAR-T: Chimeric antigen receptor-T cell
ICANS: Immune effector cell associated neurotoxicity syndrome
EEG: Electroencephalography
VE-ICANS: Visual EEG-based ICANS
AUC: Area under the curve (receiver operator curve)
ALL: Acute lymphocytic leukemia
CLL: Chronic lymphocytic leukemia
NHL: Non-Hodgkin lymphoma
CD19, BCMA: Antigens targeted by CAR-T cells
CRS: Cytokine release syndrome
CRES: Cell-related encephalopathy syndrome
MRI: Magnetic resonance imaging
CT scan: Computerized tomography
GPDs: Generalized periodic discharges
ICE score: Immune effector cell-associated encephalopathy
ASTCT: American society for transplantation and cellular therapy
LTR model: Learning-to-rank model
LASSO: Least absolute shrinkage and selection operator
CV: Cross validation
PDR: Posterior dominant rhythm
GRDA: Generalized rhythmic delta activity
LPDs: Lateralized periodic discharges
LRDA: Lateralized rhythmic delta activity
